# Pathogen profiling of inflammatory eye diseases with metagenomic deep sequencing in a tertiary referral center

**DOI:** 10.21203/rs.3.rs-11107341/v1

**Published:** 2026-10-22

**Authors:** Joanne Thomas, Kirsten Da Silva, Cindi Chen, Lina Zhong, YuHeng Liu, Thomas Abraham, Danny Yu, Armin Hinterwirth, Sruthi Arepalli, Steven Yeh, Thuy Doan, Jessica Shantha

## Abstract

Background Intraocular inflammation is a leading cause of blindness globally, but the precise etiology may be unknown in many cases due to challenges with traditional diagnostic modalities. Metagenomic deep sequencing (MDS) is an unbiased method for pathogen detection and may provide advantages over conventional diagnostic methods. Methods Patients with possible infectious eye diseases were enrolled at the Emory Eye Center from January 2018 to March 2021 for MDS analysis of intraocular fluid samples in addition to standard-of-care conventional testing, including polymerase chain reaction and bacterial and fungal cultures. Samples were sent to the Proctor Foundation at the University of California, San Francisco for analysis. Pathogens were identified through a rapid computational pipeline that analyzed non-host sequences. Data were collected on patient demographics, medical history, ophthalmic exam, and conventional diagnostic results and summarized with respect to MDS findings. Results Forty-one patients were included in this study with 25 females (61.0%) and a mean age of 55.3 years (SD:±16). The anatomic distribution of uveitis included anterior uveitis in 13 patients (31.7%), anterior/intermediate uveitis in 7 patients (17.1%), posterior uveitis in 4 patients (9.76%), panuveitis in 15 patients (36.6%), and scleritis in 2 patients (4.9%). Intraocular fluid was collected from 25 aqueous humor samples (61.0%) and 16 vitreous samples (39.0%). MDS identified pathogens in 11 of 41 samples (26.8%). The MDS positivity results by the site of sampling were 20.0% (5/25) from the anterior chamber and 37.5% (6/16) from the vitreous cavity. Pathogens identified included bacterial species such as *Klebsiella pneumoniae* , *Serratia marcescens* , and *Streptococcus pneumoniae* ; viral pathogens including Herpes Simplex Virus (HSV) and Varicella Zoster Virus (VZV); fungal organisms such as *Candida parapsilosis* and *Cladosporium* species; and parasites including *Toxocara canis.* Seven patients had an infectious etiology determined by MDS that was not detected by conventional diagnostics. Intraocular and systemic testing was concordant with MDS results in 4 patients. Conclusion Infectious organisms associated with inflammatory eye diseases presenting to a tertiary referral center are diverse and include fungi, bacteria, parasites, and viruses. The unbiased nature of MDS may be advantageous in these tertiary settings, assisting in diagnostics and guiding appropriate therapies.

